# Functional ovarian reserve in women with Sickle Cell disease: A systematic review

**DOI:** 10.5935/1518-0557.20240067

**Published:** 2024

**Authors:** Caroline Santos Silva, Nathália Muraiviechi Passos, Anna Paloma Martins Rocha Ribeiro, Raquel Rodrigues Mattos, Evangelista Torquato, Eduardo de Paula Mirada, José Bessa Junior

**Affiliations:** 1 Public Health Department of State University of Feira de Santana, UEFS, Feira de Santana, Bahia, Brazil; 2 Postgraduate Program in Human and Experimental Pathology at the Federal University of Bahia/Fundação Oswaldo Cruz, Salvador, Bahia, Brazil; 3 Christus University Center (UNICHRISTUS), Fortaleza, Ceará, Brazil; 4 Evangelista Torquato Clinic - Sollirium Health Group, Fortaleza, Ceará, Brazil

**Keywords:** Sickle cell disease, sickle cell anemia, fertility, ovarian reserve, antimullerian hormone

## Abstract

With the improvement in survival and the reduction in morbidity related to sickle cell disease (SCD), aspects related to reproductive health are emerging as a priority in the care of affected people. We conducted a systematic review looking for evidence describing the functional ovarian reserve levels in women with sickle cell disease. To locate studies, a search was performed in electronic databases, in addition to preprint servers and reference lists of selected publications. Two independent reviewers searched, and the risk of bias in the selected studies was assessed using the Newcastle-Ottawa scale. 1,086 records were initially retrieved, and one article was identified after consulting the reference lists of the screened articles, only four articles met the eligibility criteria. The quality of evidence was rated very low due to the design of the studies; however, the risk of bias was considered low. These are recent studies published between 2015 and 2021, whose main methodology was a cross-sectional or case-control study. All studies reported lower anti-mullerian hormone levels in women with sickle cell disease. Although lower, anti-mullerian hormone levels were within the normal range in young women with sickle cell disease only with supportive care, in those who used hydroxyurea, a decrease in ovarian reserve was observed. support insufficient evidence support a causal relationship between sickle cell disease and reduced ovarian reserve. From anti-mullerian hormone values a trend towards lower levels in women with sickle cell disease compared to healthy women, as reported in the four studies evaluated. Studies that analyze the ovarian reserve based on imaging and biochemical parameters are an important future focus.

## INTRODUCTION

Sickle cell disease (SCD) is a generic term that defines a group of hereditary diseases including sickle cell anemia (SCA), hemoglobin C (HbC), hemoglobin D (HbD), and beta-thalassemia, generating combinations symptomatic, called: SC hemoglobinopathy (HbSC), SD hemoglobinopathy (HbSD) and S/beta-thalassemia (HbS-β-thal), characterized by a mutation in the gene encoding the hemoglobin β subunit, resulting in the formation of abnormal sickle-shaped red blood cells (Benenson & Porter, 2018; Kato *et al.*, 2018).

The condition affects more than 100,000 people in the United States and 20 million people worldwide, being recognized by the World Health Organization (WHO) as a global public health problem (World Health Assembly, 2006).

With improvements in survival and reduction in SCD-related morbidity, aspects related to reproductive health have become a priority in the care of affected people (Chaturvedi & DeBaun, 2016). The most common reproductive problems are: delay in sexual maturation, sexual dysfunctions (Ferreira & da Silva, 2010; Smith-Whitley, 2014a, 2014b), high-risk pregnancy, complications in childbirth, and puerperium (Kuo & Caughey, 2016), limitations related to contraception measures (De Sanctis *et al.*, 2020), reduced ovarian reserve (Kopeika *et al.*, 2019) and early menopause (Ghafuri *et al.*, 2017).

The term “ovarian reserve” is often used to describe primordial follicles’ quantity and quality (European Society for Human Reproduction and Embryology (ESHRE) Guideline Group on POI *et al.*, 2016) (The ESHRE Guideline Group on POI, 2016). It has been suggested that true ovarian reserve should be the term for the pool of resting follicles (primordial follicles) (Findlay *et al.*, 2015), and that the growing pool would be better defined as the functional ovarian reserve (Moolhuijsen & Visser, 2020). According to Nelson (2014), all available markers, including quantification of the number of antral follicles by ovarian ultrasound and measurement of anti-Mullerian hormone (AMH), are a measure of “ovarian response” or “ovulatory potential” and not a measure of “ovarian reserve”, considering that at the time of measurement, it is the antral follicles that are detected, which are functionally capable of reaching the ovulatory state within a defined period of time (relatively short), given the correct stimulation of gonadotropins (Findlay *et al.*, 2015).

AMH is produced by the granulosa cells of the preantral and small antral follicles in the ovaries over the years (Fanchin *et al.*, 2003). Age-specific AMH levels vary in women from different countries (Bleil *et al.*, 2014), in addition, differences have been described between genetic factors (Shahrokhi *et al.*, 2018), ethnicities (Tal & Seifer, 2013; Kotlyar & Seifer, 2021), lifestyles and environmental exposures (Shahrokhi *et al.*, 2018).

Functional ovarian reserve can be indirectly measured by measuring biomarkers (AMH, estradiol (E2) and follicle-stimulating hormone (FSH) or ovarian ultrasonography (antral follicle count (AFC) and mean ovarian volume) (ESHRE Guideline Group on Female Fertility Preservation *et al.*, 2020; Practice Committee of the American Society for Reproductive Medicine, 2020). Although many ovarian response tests are currently being used in clinical practice, AFC and serum AMH levels have been shown to be the most promising markers, mainly due to their low intercycle variation and ease of measurement (Dewailly *et al.*, 2014).

Assessment of functional ovarian reserve is essential to assess Premature Ovarian Insufficiency (POI) in women living with SCD. Studies have raised the hypothesis that, just as iron overload in female patients with SCD results in gonadal dysfunction (Chang *et al.*, 2011; Uysal *et al.*, 2017), frequent episodes of intravascular sickling, vascular occlusion and infarction, as well as tissue hypoxia in different tissues, including the ovaries, associated with chronic anemia, could explain ovarian dysgenesis and, therefore, premature ovarian insufficiency in women with SCD (Chase *et al.*, 2009; Kopeika *et al.*, 2019).

However, it is not entirely clear how the disease and its associated factors affect functional ovarian reserve markers. In 2020, the Centers for Disease Control and Prevention (CDC)(2024) and Foundation for Women and Girls *with Blood Disorders* convened a panel of experts to assess knowledge gaps in the reproductive health of women with SCD, and among the issues listed in the final report is the need to investigation into the extent to which SCD and its treatments affect ovarian reserve. Furthermore, the authors also emphasize the importance of more studies that true impact of SCD and its treatments on fertility potential, as a way of supporting the clinical practice of counseling and guidance on fertility preservation of these women (Pecker *et al.*, 2021).

To better understand these aspects, we carried out a systematic review looking for evidence that described the levels of functional ovarian reserve in women with sickle cell disease.

## MATERIALS AND METHODS

### Type of study and research question

We performed a systematic review according to the recommendations of the “Handbook Cochrane” (Higgins *et al.*, 2019), “Methodological Guidelines: Preparation of systematic review and meta-analysis of observational studies” (Brazil, Ministério da Saúde - Secretaria de Ciência, Tecnologia e Insumos Estratégicos, 2014) and PRISMA (Page *et al.*, 2021). The research protocol was previously registered in the International prospective register of systematic reviews - PROSPERO, under the registration number CRD42023432505 (https://www.crd.york.ac.uk/prospero/display_record.php?ID=CRD42023432505).

In order to find searchable keywords that represented the clinical question of interest, the research problem was outlined (Table 1) according to the components of the acronym PICOS/PECOS, where each letter represents a component of the question, namely: population, intervention/exposure, comparison, results - outcomes and types of studies.

**Table 1:** Outline of the research problem, 2023.

P	adult patients (over 18 years old)
I/E	diagnosis of sickle cell disease (all genotypes)
C	women without a diagnosis of sickle cell disease
O	laboratory measurement of AMH and/or antral follicle count via transvaginal or transabdominal ultrasound to assess ovarian reserve
S	cohort, case-control, cross-sectional study, and clinical trials

### Databases and search strategy

A detailed literature search was performed using electronic databases: MEDLINE (via PubMed), Science Direct, LILACS, SciELO, and CAFe. Ongoing studies on the International Clinical Trial Registry Platform (ICTRP, www.who.int/ictrp) and ClinicalTrials.gov were searched. Preprint servers including MedRexiv and BioRxiv were also queried. To maximize the scope of the research, the reference lists used in all original articles and reviews on the subject were manually revised.

The search strategy combined DeCS/MeSH terms and synonyms such as: “Sickle Cell Anemias”, “Sickle Cell Disease”, “Fertility”, “Ovarian reserve”, “Ovarian follicle”, “Anti Mullerian Hormone”, “Antimullerian Hormone”. The search strategies used in the databases, as well as the findings regarding the search in the reference lists of the included articles are presented in Appendix A.

### Selection of studies

Relevant original studies that used methodologies such as: cohort, case-control, cross-sectional study, and clinical trials, regardless of publication status, were eligible for inclusion. There were no restrictions on the languages and date of publication.

Search results from the different databases were imported and incorporated into the Mendeley Reference Manager, and duplicates were excluded. Two reviewers independently assessed the retrieved titles and abstracts to identify potential articles for inclusion in this study. Then both reviewers reviewed the completed documents.

Each researcher independently extracted the following data from the included studies: author, year, country, objective, type of study, population - sociodemographic and clinical characteristics of the participants, sample size, AMH reference value to assess ovarian reserve, analysis technique of the AMH, AMH value in the studied sample and main results.

All disagreements were resolved through discussions until a consensus was reached. When consensus could not be reached between the two reviewers, the opinion of a third reviewer (supervisor) was requested, until consensus was reached.

### Evaluation of the Quality of Studies

Two reviewers independently assessed the included studies using the Newcastle-Ottawa Risk of Bias (NOS) tool (Wells *et al.*, 2012). The NOS includes separate endpoints for case-control studies and cohort studies covering the following domains: the selection of participants, comparability of study groups, and verification of exposure (for case-control studies) or outcome of interest (for cohort studies). For cross-sectional studies, the adapted NOS was used (Herzog *et al.*, 2013).

Disagreements between review authors about the risk of bias in specific studies were resolved by discussion, with the involvement of the third reviewer.

### Data extraction and presentation

After the final selection of the articles, the data were extracted in a Microsoft Excel spreadsheet and presented in a qualitative summary table of the articles specially designed to summarize the main characteristics of the studies.

## RESULTS

**Figure 1:**
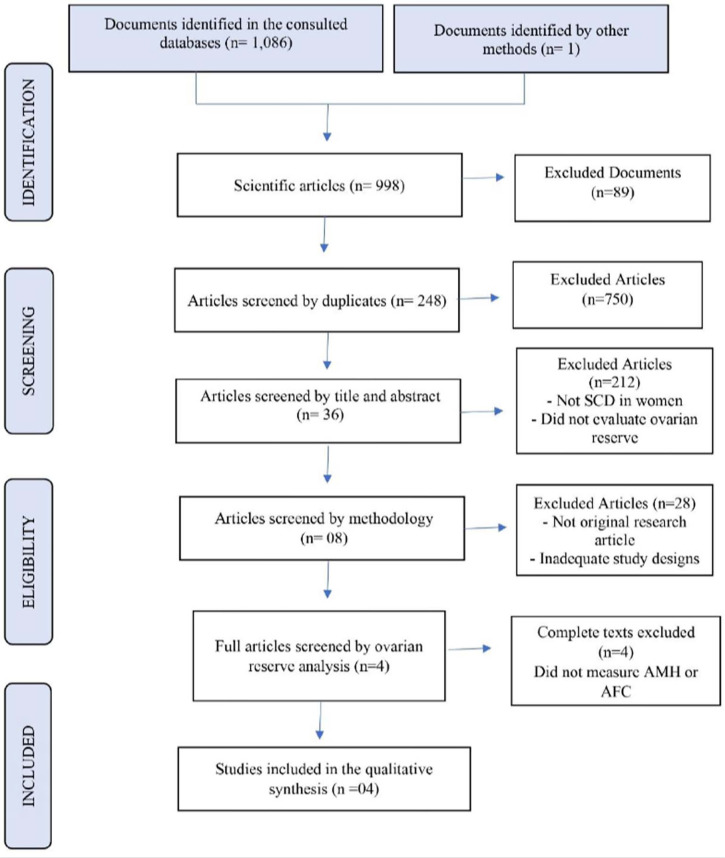
Preferred Reporting Items of Systematic Reviews and Meta-Analyses (PRISMA) flow chart of the study selection process.

The search in electronic databases resulted in 1,086 records, one article was identified after consulting the reference lists of the selected articles. After removing texts that were not scientific articles (books, chapters, editorials), 998 texts remained for analysis. After analyzing the titles and abstracts, 36 studies were submitted to a full-text review. Of these, 28 articles were excluded for inappropriate methodological aspects: they followed a methodology that was not contemplated in this study, had children as a target population, used other methodologies to assess fertility (eg, number of previous pregnancies), or reported the same cohort of another article. Finally, four studies were submitted for analysis (Figure 1).

### Characteristics of the included studies

Table 2 shows the characteristics of the studies included in this review. As for methodology, three cross-sectional studies and one case-control study were analyzed, including a total of 229 adult women with SCD (HbSS, HbSC, and HbSβ). Two studies included a control group, totaling 156 women without SCD, matched by age and/or race. The studies were conducted in the US, UK, and Nigeria, in addition to multicenter data referring to the *Multicenter Study of Hydroxyurea* (MSH) study (Pecker *et al.*, 2020) Two assays were used to measure the AMH. In 3 studies, the second-generation enzyme-linked immunosorbent assay (ELISA), with intraand inter-assay coefficients of variation of 4% and 5.3% and 4.2% and 6%, respectively. One study used an electrochemiluminometric assay (ECLIA), with intra and inter-assay coefficient of variation of 9.3 and 12.9%.

**Table 2:** Qualitative synthesis of studies included in the systematic review.

Author/year (country)	Objective	Method			AMH				Main results
		Study design	Population	Sample	Reference value	Normal	Reduced	Very low	
Elchuri *et al*., 2015 (USA)	To describe AMH and FSH levels in female SCD treated with supportive care (SC), hydroxyurea (HU) and transplantation (HSCT).	Cross- sectional	Women (HbSS or Hb Sβ Thalassemia) Age between 10-21 years old	N=76 Grouped according to SCD therapy: -supportive care (14) -receiving HU (33) -performing HSCT(09)	Normal:≥5° percentile^*^. Reduced:<5° percentile. Very low: <5° percentile and FSH >40IU/L	SC=100% HU=75.8% HSCT=0.0%	SC=0.0% HU=24.2% HSCT=11.1%	SC=0.0% HU=0.0% HSCT=88.9%	The adult patient treated with HU had reduced ovarian reserve while the adult patient undergoing HSCT had premature ovarian failure, with FSH levels at menopause.
Kopeika *et al*., 2019 (United Kingdom)	Assess how ovarian reserve and/or iron overload in SCD women could contribute to gonadal dysfunction/ovarian reserve	Control Case	Women (all genotypes) Age between 25-45 years old	N=123 50 cases 73 controls Matching: (age, ethnicity, regular cycles, AMH measurement)	Negligible: <1.5 pmol/l reduced: 1.5 to 6.5 pmol/l normal 6.6 to 19.8 pmol/l; high:>19.8 pmol/l)	SC=45% Cont.=60%	SC=28% Cont.=25%	SC=27% Cont.=15%	SCD patients of reproductive age had significantly lower levels of AMH (7.6 vs. 13.4pmol/l, *p*=0.01), which may be indicative of reduced ovarian reserve. SCD patients were significantly more likely to have lower AMH compared to the control group (OR 2.6 (CI 1.1-6.5, *p*=0.02), adjusted for HU (16% use HU). HAM also showed no correlation with HU treatment and age.
Pecker *et al*., 2020 (Multicenter)	To test the hypotheses that (1) women participating in the Multicenter Study of Hydroxyurea (MSH) would have lower AMH levels than ageand sex-matched reference values and (2) that HU exposure is associated with lower levels from AMH	Cross- sectional	Women (HBSS) adults	N=93 HU = 86 CS=07	Normal ≥1ng/ml Decreased ovarian reserve <1ng/ml	---	---	---	AMH is normal in young women without exposure to HU, although lower than reference values for women of the same age. There is a trend towards higher AMH levels in subjects randomized to placebo compared to HU; this difference was significant at ages 30 to 35 years (mean AMH 1.23 *vs*. 0.87 ng/ml, *p*<0.001) and 40 to 46 years (median AMH 0.045 vs. 0.05 ng/ml, *p*=0<005). HbSS genotype and HU therapy are risk factors for premature ovarian aging and reduced reproductive life expectancy
Garba *et al*., 2021 (Nigeria)	To determine and compare the ovarian reserve of Nigerian women with and without SCD treated at a University Hospital	Cross- sectional	Women (HbSS) Age between 18-45 years and regular menses at the time of the study.	N=166 83 cases (Pairing: age) Controls: 83 (HbAA)	Low (0.15 - 1.14ng/ml) Normal (1.15-2.56ng/ml) High (>2.65ng/ml)	SC=16.9% (High: 61.4%) Cont.=6.0% (High: 83.1%)	SC= 21.7% Cont.= 0.8%		Mean AMH was significantly lower in women with HbSS (3.64 ± 0.65 ng/ml *vs* 7.35±1.19 ng/ml, *p*<0.001). After controlling for possible confounding variables (such as age and BMI), the serum AMH level was about 4.42 ng/mL lower among HbSS participants compared to HbAA participants (adjusted β = -4.42, 95% CI: -5.88 to -2.96, *p* value <0.001). Women with HbSS demonstrated decreased ovarian reserve when compared with age-matched women with HbAA.

* SC = Women receiving supportive care only - no use of HU and HSCT

* The b5th percentile AMH concentrations for age-matched controls were classified as having low AMH: 1.07 pmol/L (0.15 ng/mL) for 10-12 years, 3.14 pmol/L (0.44 ng/mL) mL) for 13-15 years, 9.21 pmol/L (1.29 ng/mL) for 16-18 years, and 5.07pmol/L (0.71 ng/mL) for 19-21 years.

Most studies found that women’s average/median age ranged from 30 to 40, with the study by Elchuri *et al.* (2015) reporting younger women (18 to 21 years old). Only the study by Kopeika *et al.* (2019) reported the ethnicity of the participants (100% black).

Three studies described the body mass index (BMI) of the participants (cases and controls, when applicable), all of whom had a BMI <30kg/m^2^. As for harmful habits, two studies reported smoking data (Kopeika *et al.*, 2019; Garba *et al.*, 2021), and one study reported the consumption of alcoholic beverages (Garba *et al.*, 2021). The use of recreational drugs was not described in the studies.

The use of contraceptives was an exclusion criterion for the study by Garba *et al.* (2021); Kopeika *et al.* (2019) discuss this point as a limiting factor of the study, because although the controls were not using it because they had been trying to get pregnant for a year, this variable was not investigated among the cases; Elchuri *et al.* (2015) and Pecker *et al.* (2020) do not cite this information.

Garba *et al.* (2021) described the history of previous pregnancies (14 cases and 19 controls had one or more previous pregnancies), Elchuri *et al.* (2020) and Kopeika *et al.* (2019) used this variable as an exclusion criterion, while Pecker *et al.* (2020) did not address gestational aspects.

### Relationship between AMH and SCD

All studies reported lower levels of AMH in women with SCD when compared to reference values for age-matched women without the disease.

Although lower, AMH levels were in the normal range in young women with SCD with supportive care alone. In those who used hydroxyurea (HU), a more significant decrease in ovarian reserve was observed; and in the participant undergoing hematopoietic stem cell transplantation (HSCT), AMH levels indicated POI (undetectable AMH) (Elchuri *et al.*, 2020).

Kopeika *et al.* (2019) reported that women with SCD had a significantly greater chance of having lower AMH values compared to the control group (women without SCD, matched by age, ethnicity and regular cycles) (OR 2 .6 (CI 1.1-6.5, *p*=0.02), adjusted for HU (16% use HU); while Garba *et al.* (2021) described serum levels of AMH around 4.42 ng/mL lower among participants with SCD compared with controls, after adjusting for age and BMI (adjusted β= -4.42, 95% CI: -5.88 to -2.96, *p* value <0.001). Despite including participants using HU, the authors did not consider this variable in the adjustment model.

Unlike the previously cited studies, the sample by Pecker *et al.* (2020) was mainly composed of women exposed to HU (86/93). Mean AMH levels were lower in the study women than ageand sex-matched baseline values. Mean AMH levels consistent with diminished functional ovarian reserve, a risk factor for infertility, occurred in participants aged 25 to 30 years; in healthy women, this occurs after age 40. In multivariate analysis, therapeutic use of HU was independently associated with low AMH (β=0 001, 95%CI 0.002 to 0.000; *p*=0.006).

No study has evaluated the relationship between different types of hemoglobinopathies and other treatments for complications of the disease, including non-steroidal anti-inflammatory drugs (NSAIDs) and opioids and AMH levels, despite the known impact of frequent use of these medications on functional ovarian reserve.

### Other ovarian reserve markers

Only one study assessed FSH levels. Elchuri *et al.* (2015) demonstrated that women with supportive care and using hydroxyurea had FSH levels <40U/L, while the participant undergoing HSCT had FSH>40U/L, characteristic of menopause. AFC, luteinizing hormone (LH), and Estradiol or Inhibin B were not reported in the studies included in this review.

### Quality of studies

The quality of evidence was rated low due to the study design and internal and external validity features, including the risk of bias.

**Figure 2:**
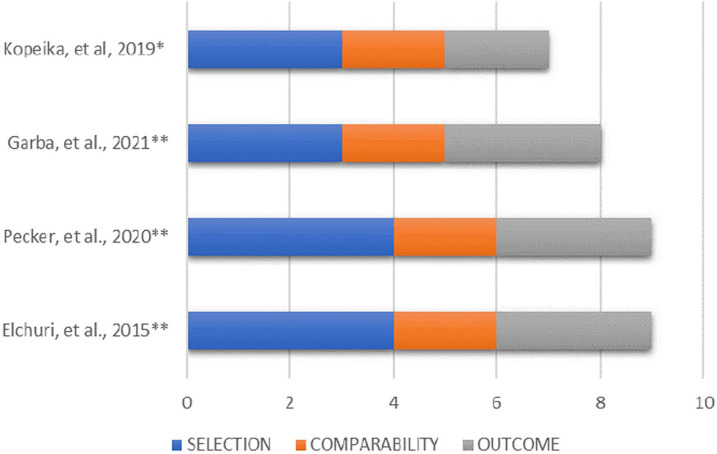
Risk of bias assessment based on the Newcastle-Ottawa scale.

The risk of bias, as assessed using the Newcastle-Ottawa scale (Wells *et al.*, 2012) and its adapted version for assessing cross-sectional studies (Herzog *et al.*, 2013), showed slight variation between studies. Scores ranging from 8/10 to 9/10 were assigned to cross-sectional studies and 7/9 to case-control studies. The sample size and lack of description of non-respondents were common limitations among studies (Figure 2).

* Study evaluated using the NOS scale (Score from 0 to 9 - the higher the score, the lower the risk of bias) (Wells et al., 2012).

** Studies evaluated using the NOS scale adapted for cross-sectional studies (Score from 0 to 10 - the higher the score, the lower the risk of bias) (Herzog et al., 2013).

## DISCUSSION

This systematic review did not find strong evidence to support a relationship between SCD and changes in functional ovarian reserve markers. Still, women with SCD have reduced levels of AMH when compared to healthy women. This discrepancy may be a result of Methodological issues arise from relatively small samples and non-community controls, as well as the need for more description of the participants’ clinical and sociodemographic characteristics.

The initial published studies evaluating fertility in women with SCD considered the number of pregnancies as the main outcome measure, and found that women with SCD had lower rates of reported pregnancies during their reproductive years (Jimenez *et al.*, 1966; Alleyne *et al.*, 1981). This parameter is inappropriate to evaluate the fertility potential in this population, as many other factors may have influenced the number of pregnancies per patient, such as lower rates of sexual activity (Samuels-Reid *et al.*, 1984) disease-modifying treatment toxicity Eissa *et al.* (2015).

Currently, AMH is considered the most accurate predictor of ovarian reserve, though fluctuations may occur as a result of ethnic differences, BMI, smoking, among other. However, these oscillations appear to be random and minor, thus allowing the measurement of AMH regardless of cycle phase (Dewailly *et al.*, 2014). For Cedars (2022), AMH levels must be interpreted in the context of the endogenous endocrine environment, where low FSH, due to hypogonadotropic hypogonadism or the use of hormonal contraceptives, may decrease AMH without being a true reflex of functional ovarian reserve. Furthermore, there is an inverse correlation between BMI and AMH that does not reflect functional ovarian response. Therefore, It is usually recommended that other measures, such as AFC, ovarian volume, estradiol or inhibin B, and FSH should be obtained in combination with AMH to allow for more definitive conclusions.

In our systematic review there was also considerable heterogeneity in the analysis and description of the AMH. Such aspects made it impossible to synthesize the data with a meta-analysis. Standardizing AMH assessment/description is of great importance for advancing the knowledge in the population of women with SCD. Also, it is desirable that more studies providing a more robust evaluation of ovarian function in women with SCD be performed. The diagnostic role and clinical relevance of the apparent reduction in AMH in women with SCD have yet to be established. Studies that analyze the functional ovarian reserve based on imaging and biochemical parameters are an important future focus and should play a prominent role in studies assessing fertility aspects in this population.

Women with transfusion-dependent SCD have significantly lower levels of FSH, LH, estrogen (Skillern & Rajkovic, 2008), and AMH (Chang *et al.*, 2011). Similarly, those undergoing HSCT had undetectable AMH and FSH levels corresponding to menopause, suggesting profound ovarian insufficiency. It is suggested that alkylators and HU have an additive gonadotoxic effect (Elchuri *et al.*, 2015). Among the included studies, only (Elchuri *et al.*, 2015) evaluated women undergoing blood transfusion, while Kopeika *et al.* (2019) and Garba *et al.* (2021) considered this an exclusion factor for their studies. As for HSCT, this was also an exclusion criterion for the studies, except for Elchuri *et al.* (2015). The heterogeneity of the inclusion criteria further limits our findings’ interpretations and causal relationships.

The negative potential on fertility and the occurrence of hypogonadism associated with the use of opioids and NSAIDs is known. Despite the drugs used to treat chronic pain in SCD, studies have shown a reduction in hormone levels in women using opioids (Daniell, 2008) and NSAIDs (Jesam *et al.*, 2014). However, little has been described about the potential for infertility of these drugs in women with SCD. Although it is a known fact that opioids suppress the hypothalamic-pituitary-gonadal axis by inhibiting the release of gonadotropin-releasing hormone (GnRH) (Seyfried & Hester, 2012; Gudin *et al.*, 2015), resulting in reduced LH levels and disruptions in the menstrual cycle (Santen *et al.*, 1975), the exact mechanisms though which these medications lead to decreased AMH is not defined.

Finally, it is important to mention that the role of psychosocial stress on functional ovarian reserve has also not been evaluated among the women studied. Chronic psychosocial stressors, depressive symptoms, and stress have been shown to be detrimental to the functional ovarian reserve in healthy women aged between 25 and 45 years (Bleil *et al.*, 2012). Although these experiences are common for people with SCD (Pecker & Darbari, 2019; Al-Marzouki *et al.*, 2021), it seems very important to investigate the intersection between SCD and these factors in future research.

In this context, women with SCD, who are still of reproductive age, on uninterrupted hydroxyurea therapy due to severe SCD, or are expected to undergo HSCT or gene therapy, should be referred to a reproductive endocrinologist to discuss fertility preservation, including several, such as ovarian transposition, oocyte and embryo cryopreservation, and ovarian cryopreservation. These options have the potential to impact quality of life definitely (Ghafuri *et al.*, 2017).

## CONCLUSIONS

Women with SCD have lower levels of AMH when compared to healthy women. As this condition exposes women to many risks and uncertainties, studies with more robust designs are needed to evaluate the association between sickle cell disease and premature ovarian failure.
